# Arbitrary methodological decisions skew inter-brain synchronization estimates in hyperscanning-EEG studies

**DOI:** 10.1162/imag_a_00350

**Published:** 2024-11-01

**Authors:** Marius Zimmermann, Kathrine Schultz-Nielsen, Guillaume Dumas, Ivana Konvalinka

**Affiliations:** Chair of Cognitive Neuroscience, Institute of Psychology, University of Regensburg, Regensburg, Germany; Section for Cognitive Systems, DTU Compute, Kongens Lyngby, Denmark; CHU Sainte-Justine Research Centre, Department of Psychiatry, Université de Montréal, Montréal, QC, Canada; Mila – Quebec AI Institute, Montréal, QC, Canada

**Keywords:** hyperscanning, inter-brain synchronization, EEG, circular correlation, epoch length, signal-to-noise ratio

## Abstract

Over the past decade, hyperscanning has emerged as an important methodology to study neural processes underlying human interaction using fMRI, EEG, fNIRS, and MEG. However, many methodological decisions regarding preprocessing and analysis of hyperscanning data have not yet been standardized in the hyperscanning community, yet may affect inter-brain estimates. Here, we systematically investigate the effects common methodological choices can have on estimates of phase-based inter-brain synchronization (IBS) measures, using real and simulated hyperscanning (dual) EEG data. Notably, we introduce a new method to compute circular correlation coefficients in IBS studies, which performs more reliably in comparison to the standard approach, showing that the conventional circular correlation implementation leads to large fluctuations in IBS estimates due to fluctuations in circular mean directions. Furthermore, we demonstrate how short epoch durations (of 1 s or less) can lead to inflated IBS estimates in scenarios with no strong underlying interaction. Finally, we show how signal-to-noise ratios and temporal factors may confound IBS estimates, particularly when comparing, for example, resting states with conditions involving motor actions. For each of these investigated effects, we provide recommendations for future research employing hyperscanning-EEG techniques, aimed at increasing validity and replicability of inter-brain synchronization studies.

## 1 General Introduction

The past decade has seen a major rise in hyperscanning studies, with a particular focus on measuring synchronized brain activity across interacting participants, so-called inter-brain synchronization (IBS) (F. Babiloni & Astolfi, 2014;Czeszumski et al., 2020;Dumas, 2011;Konvalinka & Roepstorff, 2012). Hyperscanning is a methodology for measuring brain activity simultaneously in multiple participants either using electroencephalography (EEG) (C. Babiloni et al., 2006;Bevilacqua et al., 2019;Dumas et al., 2010;Endevelt-Shapira et al., 2021;Goldstein et al., 2018;Kayhan et al., 2022;Konvalinka et al., 2014;Koul et al., 2023;Leong et al., 2017;Lindenberger et al., 2009;Zamm et al., 2018), magnetoencephalography (MEG) (Ahn et al., 2018;Baess et al., 2012;Lin et al., 2023;Zhou et al., 2016), functional near-infrared spectroscopy (fNIRS) (Cui et al., 2012;De Felice et al., 2023;Jiang et al., 2012;Nguyen et al., 2021;Pan et al., 2017;Pinti et al., 2020), or functional magnetic resonance imaging (fMRI) (Bilek et al., 2022;Goelman et al., 2019;Misaki et al., 2021;Montague et al., 2002). The methodology emerged together with a call in social cognition research to move away from studies of individuals in isolation and toward studies of true interactions (Hari & Kujala, 2009;Schilbach et al., 2013), with the goal of better understanding the interpersonal and interactive mechanisms, also on a neural level (Konvalinka & Roepstorff, 2012). With increasing numbers of hyperscanning studies in recent years, numerous computational methods have been used and applied to two- (or more) brain data, and IBS in particular; however, these methods have not always been tested thoroughly, which has resulted in a multitude of analysis approaches and choices in hyperscanning pipelines, leading to a wide range of results that are often difficult to compare. Moreover, some of the commonly used methods to quantify IBS have not been thoroughly investigated, and may lead to variable results. A recently developed hyperscanning-EEG toolbox (Ayrolles et al., 2021) and practical guide (Zamm et al., 2023) provide promise in working toward a standardization of future hyperscanning-EEG pipelines; however, many of the methodological decisions which have to be made by researchers have still not been standardized, and are often arbitrarily chosen, which may lead to inconsistent results.

Within the EEG-hyperscanning literature, non-directional analyses commonly focus on alignment or synchronization of the phase of signals, using estimation of phase locking values (PLV) (Dumas et al., 2010;Pérez et al., 2017;Yun et al., 2012), phase lag index (PLI) (Ahn et al., 2018;Lindenberger et al., 2009;Sänger et al., 2013), or circular correlations (Burgess, 2013;Goldstein et al., 2018) between time series. However, even within conceptually similar tasks, a wide range of observed effects have been reported, in terms of inter-brain networks, as well as the frequency bands within which synchronization occurs. For example, some EEG-hyperscanning studies have reported IBS across theta and delta frequencies during coordinated action, for example, guitar playing (Lindenberger et al., 2009;Sänger et al., 2012); while other studies have reported effects at higher frequencies, at alpha (Dumas et al., 2010;Goldstein et al., 2018;Lin et al., 2023), beta (Yun et al., 2012), and gamma (Astolfi et al., 2010;Dumas et al., 2010) frequency ranges.

Here, we address the issue that a wide range of experimental and analytical decisions are implied by the computation of inter-brain estimates, and that these choices are likely to contribute to the variability in findings, raising concerns regarding their validity. Therefore, we systematically investigate consequences of some of the methodological decisions made when estimating IBS in EEG studies, using both simulated and real (dual-EEG) data (fromZimmermann et al., 2022). Specifically, we investigate: the calculation of circular correlation for continuous signals, as a common measure to quantify IBS; how the choice of epoch length affects estimates of IBS, using circular correlations and phase locking values in particular; and how differences in frequency power and signal-to-noise ratio affect IBS estimates. While the focus in this paper is on dual-EEG studies, the concerns raised in this paper also apply to studies involving simultaneous recordings in more than two people, and possibly other neuroimaging techniques.

### 1.1 Circular correlation

One common procedure to estimate IBS is to calculate the circular correlation between signals from two sensors from two respective participants. This method was introduced byBurgess (2013)as an improvement to previous methods that are more prone to spurious coupling, in particular, PLV (Burgess, 2013). Burgess defines circular correlation,$p_{c}$, as:

$$p_{c}\left(\alpha ,\beta \right)=\frac{\sum \sin\left(\alpha -\mu \right)\text{sin}\left(\beta -\nu \right)}{\sqrt{\sum sin^{2}\left(\alpha -\mu \right)\sum sin^{2}\left(\beta -\nu \right)}}$$ 1.1

whereαandβrepresent the instantaneous phases at electrodes 1 and 2, andμandνrepresent the circular mean directions for electrodes 1 and 2, respectively. Hence,sin(α−μ)andsin(β−ν)represent the deviations of the two phases from their mean directions. The equation is derived fromJammalamadaka and SenGupta (2001)and implemented in the “CircStat” Toolbox (Berens, 2009). Noteworthy, the CircStat toolbox is intended for calculation of (circular) correlations between discrete events, such as wind and flight directions. This is in line with the descriptions of Jammalamadaka and SenGupta, where a correlation is calculated between*“random sample[s] of observations which are directions of two attributes”*(Jammalamadaka & SenGupta, 2001; section 8.2). However, it should be noted that according to Jammalamadaka, section 8.2, equation 8.2.2 (corresponding toequation 1.1above) is valid for cases with well-defined circular mean values. In case of arbitrary or not well-defined mean directions, such as in case of uniform distributions for the signals, the mean directions should be chosen such that they*“yield the largest possible association in both positive and negative directions”*(Jammalamadaka, 8.2(ii)), and as such maximize the positive or negative correlation. Hence, a different equation (equation 8.2.4) is required (see comment 8.2.2(ii)), resulting in an adjusted definition of circular correlation for signals with arbitrary means:

$$p_{c\_adj}=\frac{\left(R_{\alpha \;-\;\beta }-R_{\alpha \;+\;\beta }\right)}{2\sqrt{\sum sin^{2}\left(\alpha -\mu \right)\sum sin^{2}\left(\beta -\nu \right)}}$$ 1.2

with

$$R_{\alpha \;\pm \;\beta }=\left|\sum e^{i\left(\alpha \;\pm \;\beta \right)}\right|$$

In this case, the numerator of the adjusted circular correlation,$p_{c\_adj}$, becomes the difference in the lengths of the mean vectors ofα−βandα+β.

**Fig. 1.**
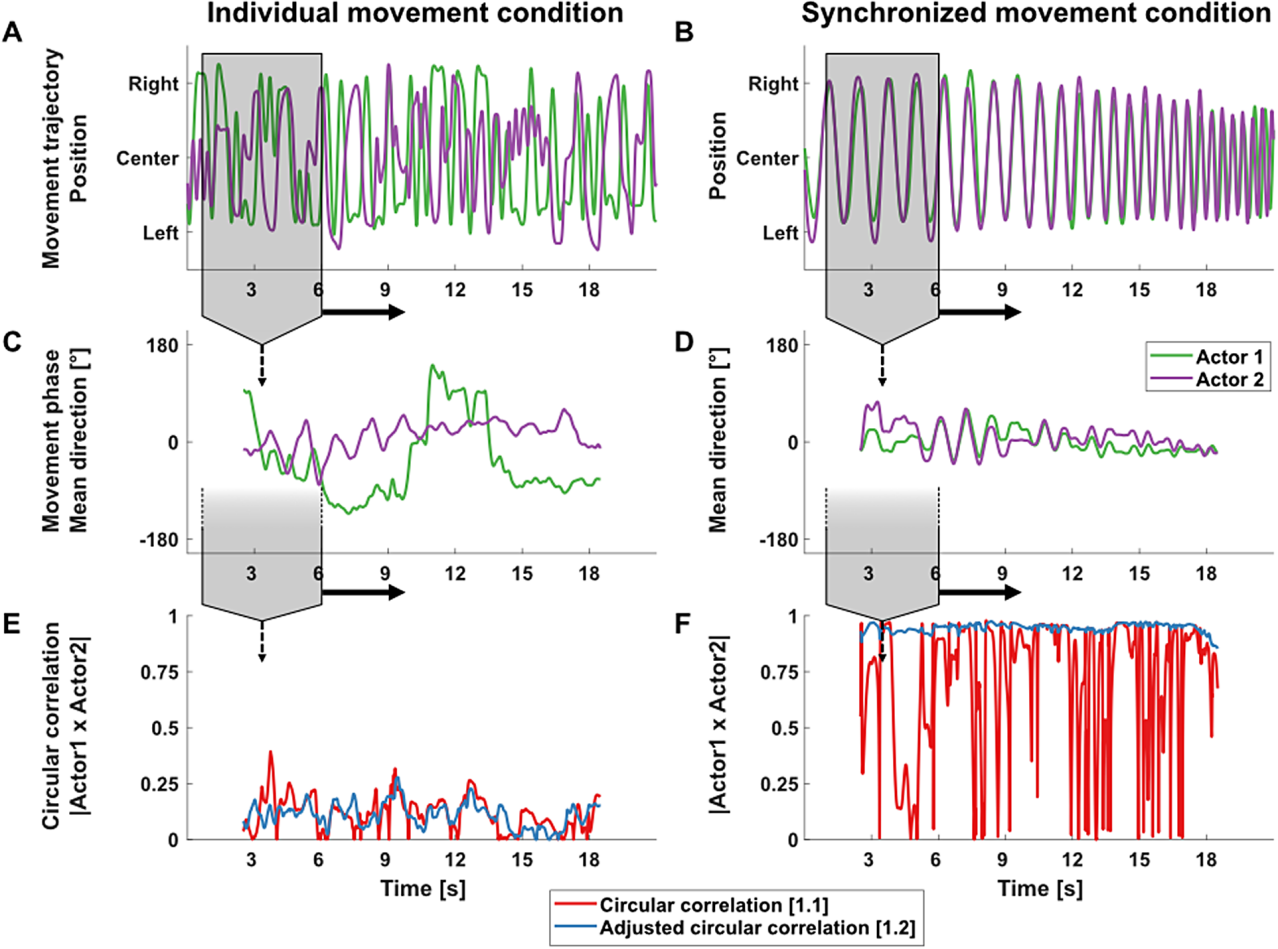
Generation and evolution of circular mean and circular correlation. Process and estimates are based on individually performed, non-synchronized movements of two actors (left column) and synchronized movements of pair of actors during the mirror game paradigm (right column). (A, B) Raw movement trajectories of two actors (red, blue). (C, D) Corresponding circular mean direction of movement phases over a 5-s moving segment (indicated in grey) for the data shown in A and B, respectively. (E, F) Circular correlation calculated for assumed non-uniform (red;equation 1.1) and assumed uniform (blue;equation 1.2) distribution of phase angles over the same 5-s moving segment for the data shown in A and B.

We argue that continuous data, such as EEG data, should be considered as data having arbitrary mean directions, as the mean direction of (arbitrarily chosen) signal segments is not well defined. This can be demonstrated using movement trajectories from two conditions of the mirror game, where pairs of participants performed either synchronized movements, or individual, non-synchronized movements (seeZimmermann et al., 2022, for details). As illustrated inFigure 1, the circular mean direction of a trajectory segment fluctuates wildly with small changes of the analysis window, and as a consequence, estimates based on the unadjusted circular correlation (equation 1.1) fluctuate. In fact, using the adjusted circular correlation (equation 1.2), the estimates are stable and correspond to the (subjective) impression of the level of synchronization between movement trajectories, which is particularly evident in the synchronized movement condition (Fig. 1F).

Thus, with respect to EEG, this would mean that the mean direction of a specific signal segment depends on the signal epoch length and position, and may change drastically with small changes (in the range of samples) in duration or on/offset. In**experiment 1**, we investigate the consequences of applyingequation 1.1andequation 1.2, using simulated and real EEG data (obtained fromZimmermann et al., 2022). We predict more stable estimates over a range of small, arbitrary changes in data processing by using the adjusted variant to calculate circular correlations.

### 1.2 Epoch length

Epoch lengths that are used to estimate inter-brain measures are often arbitrarily chosen, or standardized to 1 s (Ayrolles et al., 2021;Bevilacqua et al., 2019;Goldstein et al., 2018). In**experiment 2**, we investigate how IBS estimates depend on epoch length, both on simulated data with varying degrees of coupling, as well as real EEG data from an interactive and individual mirror game performance. Related work on intra-brain synchronization showed that PLI-based functional connectivity (intra-brain) estimates in resting-state EEG recordings decrease with increasing (low) epoch lengths, and stabilize only at epoch lengths of 6–12 s (Fraschini et al., 2016), and it has been recommended that epochs of lengths shorter than 4 s should hence be avoided where possible (Miljevic et al., 2022). Moreover, phase coupling estimates have previously been shown to be dependent on the number of cycles of oscillation present for each epoch (Basti et al., 2022), such that epoch or window lengths that are shorter result in higher and less reliable phase estimates, even for uncoupled signals. Correspondingly, we expect inflated IBS estimates at short epoch lengths, and further expect that they tend to stabilize at longer epoch lengths than those that are often used.

### 1.3 Power and signal-to-noise ratio

Phase is generally considered to be independent of signal amplitude; however, it has been suggested that phase*estimates*can be affected by signal amplitude, or power (van Diepen & Mazaheri, 2018). Whereas phase and amplitude are technically unrelated, phase estimates in weaker signals (or signals with lower amplitude/power) may be relatively more prone to noise, resulting in less stable phase estimates. This is particularly relevant for inter-brain comparisons across conditions that have different signal-to-noise ratios (SNR). For example, many hyperscanning-EEG studies have looked at inter-brain mechanisms during reciprocal movement coordination (Dumas et al., 2010;Konvalinka et al., 2014;Ménoret et al., 2014;Tognoli et al., 2007;Zimmermann et al., 2022), and contrasted such conditions of coupled interaction with uncoupled movement production (e.g., with a metronome, independent movements, etc.). Individual brain analyses have shown that coupled interactions yield the highest mu-suppression in contrast to uncoupled movements or rest (Dumas et al., 2010;Konvalinka et al., 2014;Lachat et al., 2012), corresponding to amplitude suppression of oscillations at 10 and 20 Hz over sensorimotor areas (Gastaut & Bert, 1954;Salenius et al., 1997). As amplitude suppression leads to lower SNR, and, hence, potentially a poorer ability to estimate phase from the signal, this may have an effect on the phase-based inter-brain estimates, as well as the comparisons between conditions of stronger and weaker mu suppression. Therefore, in**experiment 3**, we will investigate whether and how signal amplitude can affect estimates of inter-brain synchronization which depend on phase estimates, such as PLV and circular correlation. Additionally, we will investigate the potential consequences of such effects on comparisons of inter-brain synchronization between conditions with differences in signal amplitude, which can occur in cases of mu- or alpha-suppression.

Across these experiments, we show using both simulations and experimental dual-EEG data that inter-brain synchronization estimates are drastically affected by arbitrary decisions regarding the mean direction in circular correlations, the epoch length and epoch onset/offset in continuous signals, and short epoch lengths used to estimate phase-based IBS. Furthermore, we show how signal amplitude (i.e., power) can affect estimation of phase-based connectivity measures, which can lead to false positives or false negatives when comparing conditions with varying degrees of (social) interaction. The overall aim of this paper is, therefore, a call for an effort to set common standards for methodological decisions in hyperscanning-EEG experiments, and to provide recommendations regarding decisions that we show can have substantial effects on IBS results, with the goal of increasing the validity and reproducibility of inter-brain findings.

## 2 Methods

### 2.1 Data

#### 2.1.1 Generating artificial data

In order to generate artificial data, we used FieldTrip’s (version 20230503,Oostenveld et al., 2011) ft_connectivitysimulation function in Matlab (R2022b; The MathWorks, Natick, USA) with a known connectivity structure. Specifically, we used a linear mixing model with two observed signals and one unobserved signal, and additional independent white noise for each observed signal. Conceptually, the observed signals represent independent electrodes of two participants (with independent signals generated by independent white noise) that can be affected by a common “inter-brain” process, the unobserved signal. Therefore, the amplitude of the unobserved signal was varied systematically to generate inter-brain synchronization of varying strength, that is, imitating data from varying levels of neural interaction. Example data for the different coupling levels are shown inFigure 2A-C.

**Fig. 2.**
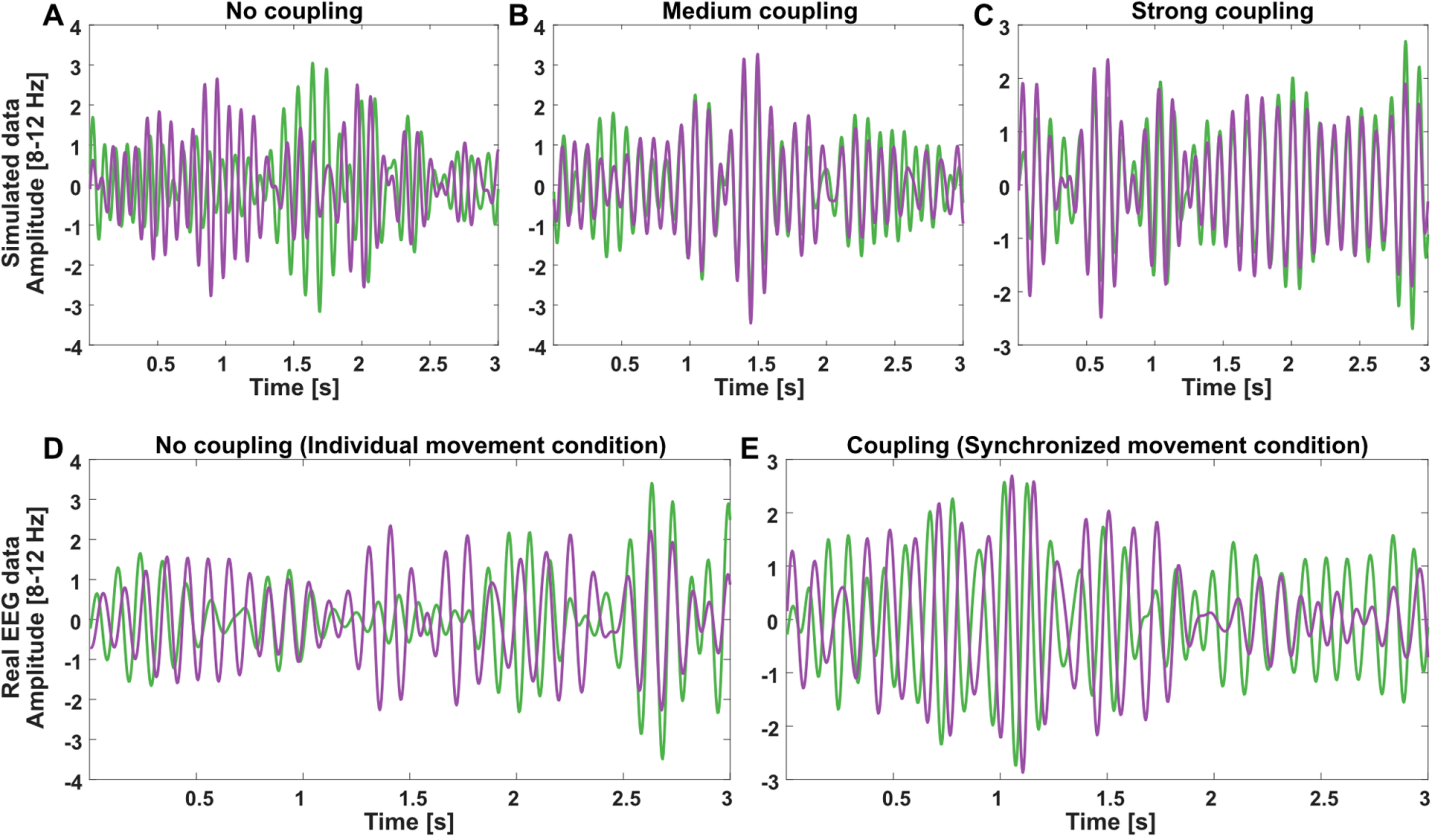
Example data. Top panels (A-C): two simulated time series with no, medium, and strong “common signal”, representing increasing coupling levels. Bottom panels (D-E): real EEG time series data from no coupling and coupling conditions (mirror game, individual and synchronized movements). Time series are band-pass filtered to the alpha frequency band (8–12 Hz), and amplitudes are normalized.

Data were generated for 100 trials at a time, with a sampling frequency of 256 Hz (matching the real data after preprocessing, see below), with trial durations of 3 s, unless noted otherwise. Additional data processing steps are specified in the corresponding sections.

#### 2.1.2 Real dual-EEG data

Real dual-EEG data were taken from a previous EEG study (Zimmermann et al., 2022). The study was conducted according to the Declaration of Helsinki and was approved by DTU Compute’s Institutional Review Board (COMP-IRB-2020-02). All participants in this study provided written informed consent for being included in the study. In this study, dyads participated in a mirror game task (Noy et al., 2011) while EEG was recorded from both participants simultaneously, using two daisy-chained 64-channel BioSemi (Amsterdam, the Netherlands) ActiveTwo systems. Participants were asked to generate, among other conditions, synchronized movements while observing each other’s hands, or generate movements individually without seeing the other person. Each condition trial lasted 25 s, out of which 21 s were analyzed (removing 2 s at the beginning and end of each segment to allow for movement synchronization to stabilize). Each condition was repeated 16 times. Data were preprocessed using band-pass filtering (1–40 Hz; two-pass 4^th^-order Butterworth filter), resampling to 256 Hz, ICA to remove eye and muscle artifacts, and re-referenced to the global average before analyses. Data from 18 dyads were segmented into segments of 3 s, which form the “trials” in this report. Per condition, the first 100 trials/segments were used in this report. For details regarding task, data recording, and preprocessing, seeZimmermann et al. (2022). Example behavioral data are shown inFigure 1, and corresponding neural data are shown inFigure 2D-E.

For the purpose of the current analyses, we assumed that data corresponding to synchronized movements show a higher coupling level (i.e., higher inter-brain synchronization) than data corresponding to individually performed movements. This assumption is based on a number of studies suggesting increased inter-brain synchronization in interacting dyads (e.g.,Dumas et al., 2010). However, we note that the focus of this report is not on synchronization itself, but the effects of arbitrary decisions on estimations of inter-brain synchronization.

#### 2.1.3 Uniform distribution of EEG data (artificial and real data)

We tested the assumption that EEG data (generated and real data) are uniformly distributed using Hodges-Ajne omnibus tests for nonuniformity (CircStat toolbox; function: circ_otest;Berens, 2009). We generated and band-pass filtered [8–12 Hz] data for 10.000 epochs using the same parameters as for EEG data generation, and tested each epoch for nonuniformity. The null hypothesis that the signal comes from a uniform distribution has been rejected in only 6 out of 10.000 cases (0.06%; p < .05, uncorrected). Thus, in >99% of generated data, the distribution is assumed to be uniform. The same approach has been applied to real data segments, using 1 s band-pass filtered [8–12 Hz] epochs for all dyads and trials, for a total of 20628 evaluated epochs. The null hypothesis was rejected in 10 out of 20628 cases (0.05%; p < .05, uncorrected); in >99% of real EEG data epochs, the distribution is assumed to be uniform. Without additional (i.e., 8–12 Hz) band-pass filtering, the null hypothesis was rejected in 19.28% of real EEG data epochs. These outcomes support the assumption that EEG data are uniformly distributed.

### 2.2 Experiment 1: circular correlation

#### 2.2.1 Data processing

For artificial data, simulated trials with durations of 3 s were generated following the general procedure described insection 2.1.1. Specifically, sets of 100 trials were generated with low individual signal amplitude (standard deviation: 0.2 [cfg.absnoise]), and no (std: 0), medium (std: 0.4), and strong (std: 0.8) “common” signal [cfg.mix], representing “coupling levels”. No delay [cfg.delay] was specified. Next, data for each trial were band-pass filtered corresponding to the alpha frequency band (8–12 Hz; two-pass 4^th^-order Butterworth filter). Example data are shown inFigure 2. Instantaneous phase angles were estimated using Hilbert transforms in Matlab, and circular correlations were estimated usingequations 1.1and1.2.

For real data, EEG signals corresponding to synchronized and individual movements of dyads were used for these analyses (seeZimmermann et al., 2022). EEG data of 3 s segments were extracted for the right lateralized, central electrode (C3) and band-pass filtered corresponding to the alpha frequency band (8–12 Hz; two-pass 4^th^-order Butterworth filter), where inter-brain synchronization has been reported in previous studies involving interpersonal motor coordination (Dumas et al., 2010;Goldstein et al., 2018), and C3 has been shown to be relevant to movement coupling in the mirror game (Zimmermann et al., 2022). To estimate circular correlations between signal segments based onequation 1.1andequation 1.2respectively, instantaneous phase angles of preprocessed EEG data were estimated using Hilbert transforms in Matlab, following the same procedures as were used for simulated data.

A Matlab script calculating adjusted circular correlations for univariate data is provided via Github (https://github.com/marizi/CCorrIBS) and can be used as an extension to the Circular Statistics toolbox (Berens, 2009), as well as an update to the circular correlation implementation in the EEG hyperscanning toolbox, HyPyP (Ayrolles et al., 2021). An implementation of the adjusted circular correlation for python is also available in the Pingouin package (Vallat, 2018;https://pingouin-stats.org/build/html/generated/pingouin.circ_corrcc.html).

#### 2.2.2 Comparison of circular correlation estimates

First, we compared the two approaches to estimate circular correlations on*average trial estimates*. For this, 1 s (256 samples) long epochs were used to estimate circular correlations usingequation 1.1. and1.2respectively, for no, medium, and strong coupling levels. Segment length was based on common choices used in the literature (Ayrolles et al., 2021;Bevilacqua et al., 2019;Dumas et al., 2010;Goldstein et al., 2018). Estimates were compared using 2-way ANOVAs with factors of approach (discrete, uniform) and coupling (artificial data: no, medium, strong; real data: individual, synchronized). Moreover, we compared circular correlation estimates (adjusted and unadjusted) for individual trials to PLV estimates for the same epochs using Pearson correlations.

Second, we investigated the effect of*onset shifting*at the sample level. For this, a 1 s (256 samples) epoch was taken from each trial, and then shifted by one sample (corresponding to less than 0.004 s) at a time. For each (shifted) epoch, circular correlation was estimated using the representative equations. A total of 512 epochs/shifts were generated using this procedure. Then, for each trial, the average absolute change in circular correlation estimates was calculated over all onset shifts, providing a single value of the average change per trial. These average changes are used as a measure of variability in circular correlation estimates with small shifts in epoch onset. It should be noted that a simple standard deviation would not be able to distinguish between gradually changing circular correlation estimates and estimates that fluctuate randomly (e.g., a permuted sequence of gradually changing estimates). Our shift-wise change measure corresponds to a root mean square of sample-by-sample changes. The averaged sample by sample changes were compared using 2-way ANOVAs with factors of approach and coupling.

Third, we investigated the effect of*epoch duration*at the sample level. Similar to the effect of onset shifting, 1-s (256 samples) epochs were taken from each trial, and then increasingly extended by one sample. For each (extended) epoch, circular correlation was estimated according to both equations. Epochs were extended up to 3 s, providing 512 epochs/extensions that were generated using this procedure. For each trial, the changes in correlation estimate with each extension were averaged over all extensions, providing a single value of the average change per simulation. These average changes are used as a measure of variability in circular correlation estimates with small changes of epoch duration, and compared using 2-way ANOVAs with factors of approach and coupling.

All analyses were performed for simulated data using three levels of coupling (random, medium, and strong), and separately for real EEG data, using data from individual movement production and interactive, synchronized movement production. For real EEG data, analyses were performed for each dyad (N = 18) individually. Dyad averages for each measure were stored for group level comparisons using 2-way (coupling level (high, low) x approach (discrete, uniform)) within-subject ANOVAs. Alpha levels for all statistical comparisons were set to p < .05.

### 2.3 Experiment 2: epoch length

#### 2.3.1 Data processing

For artificial data, simulated trials with durations of 20 s were generated following the general procedure described insection 2.1.1. Specifically, sets of 100 trials were generated with low individual signal amplitude (standard deviation: 0.2 [cfg.absnoise]) and*no*(std: 0),*medium*(std: 0.4), or*strong*(std: 0.8) “common” signal [cfg.mix], representing “coupling levels”. No delay [cfg.delay] was specified. Next, data for each trial were band-pass filtered corresponding to the alpha frequency band (8–12 Hz). Example trials are shown in the general methods section. The same data were used for each range of epoch length, spanning between 0.1 and 20 s in steps of 100 ms. For each segment, instantaneous phase angles were estimated using Hilbert transforms in Matlab. Temporal PLV (Dumas et al., 2010) and adjusted circular correlation were calculated.

Real EEG data corresponding to synchronized and individual movements of dyads were used for these analyses (seeZimmermann et al., 2022). EEG data of 20 s segments were extracted for electrode C3 and band-pass filtered corresponding to the alpha frequency band (8-12 Hz), where inter-brain synchronization has been reported in previous studies (Dumas et al., 2010;Goldstein et al., 2018). Next, segments corresponding to different epoch length ranging from 0.1 to 20 s in steps of 100 ms were selected, starting from the onset of each trial segment, corresponding to the data segments generated for artificial data. Instantaneous phase angles of preprocessed EEG data were estimated using Hilbert transforms, and temporal PLV and adjusted circular correlation were estimated, following the same procedures as were used for simulated data (see above).

#### 2.3.2 Analysis of the effect of epoch length

Following visual inspection of the data, we fitted exponential functions (b1 * exp(-b2*X) + b3) to the averages over all simulations to estimate and compare the strength of the “decay” in estimated circular correlation values for each coupling level for simulated and real data.

### 2.4 Experiment 3: power/signal to noise ratio

#### 2.4.1 Phase estimation error and SNR

Data were generated for 100 trials of 3 s at each amplitude level using ft_freqsimulation. Simulated data were generated by superimposing a 10 Hz oscillation with varying amplitude (from 0.25 to 5) in steps of 0.05, and a random noise with fixed amplitude of 1. Generated data were band-pass filtered at 8–12 Hz. Phase estimation error was calculated as the root mean square (RMS) difference between estimated instantaneous phases (using Hilbert transform, see above) between 0.5 and 2.5 s of each trial (excluding possible edge effects) based on the combined (oscillation + noise) and the clean (oscillation only) signals.

#### 2.4.2 IBS and relative noise levels

Data were generated for 100 trials of 3 s at each coupling and noise level using ft_connectivitysimulation, following the general procedures described above. The coupling level (cfg.mix) was varied from no coupling (cfg.mix = 0) to medium (cfg.mix = 0.4) and high (cfg.mix = 0.8); level of noise (cfg.absnoise) was varied from low (cfg.absnoise = 0.1) up to high (cfg.absnoise = 1.0) in steps of 0.1. PLV was calculated based on band-pass filtered (8–12 Hz) data. Statistical comparisons were performed using 2-way ANOVA with three levels of coupling (no, medium, strong) and three levels of relative noise (0.1, 0.4, 1.0).

#### 2.4.3 Power/SNR and inter-brain synchronization measures in real EEG data

EEG signals corresponding to individual movements and two 2-min rest conditions were used for these analyses (seeZimmermann et al., 2022). EEG data of 3 s segments were extracted for right lateralized, central electrode (C3) and band-pass filtered corresponding to the alpha frequency band (8–12 Hz). Inter-brain synchronization was estimated using adjusted circular correlation, and amplitude envelopes of preprocessed EEG data were estimated using Hilbert transforms in Matlab. To reduce artifacts during the rest condition, segments that had adjusted circular correlation estimates or amplitude in either dyad more than two inter-quartal ranges from the median were excluded from the analysis. We compared adjusted circular correlation estimates from a rest condition with estimates in the uncoupled (individual) condition of the mirror game, from the same dyads and recording session, using paired t-tests. Furthermore, we correlated average signal amplitude (based on the Hilbert envelope amplitude) with estimates of adjusted circular correlations.

## 3 Results

### 3.1 Experiment 1: circular correlation

#### 3.1.1 Variability of circular mean values of continuous EEG signals

**Fig. 3.**
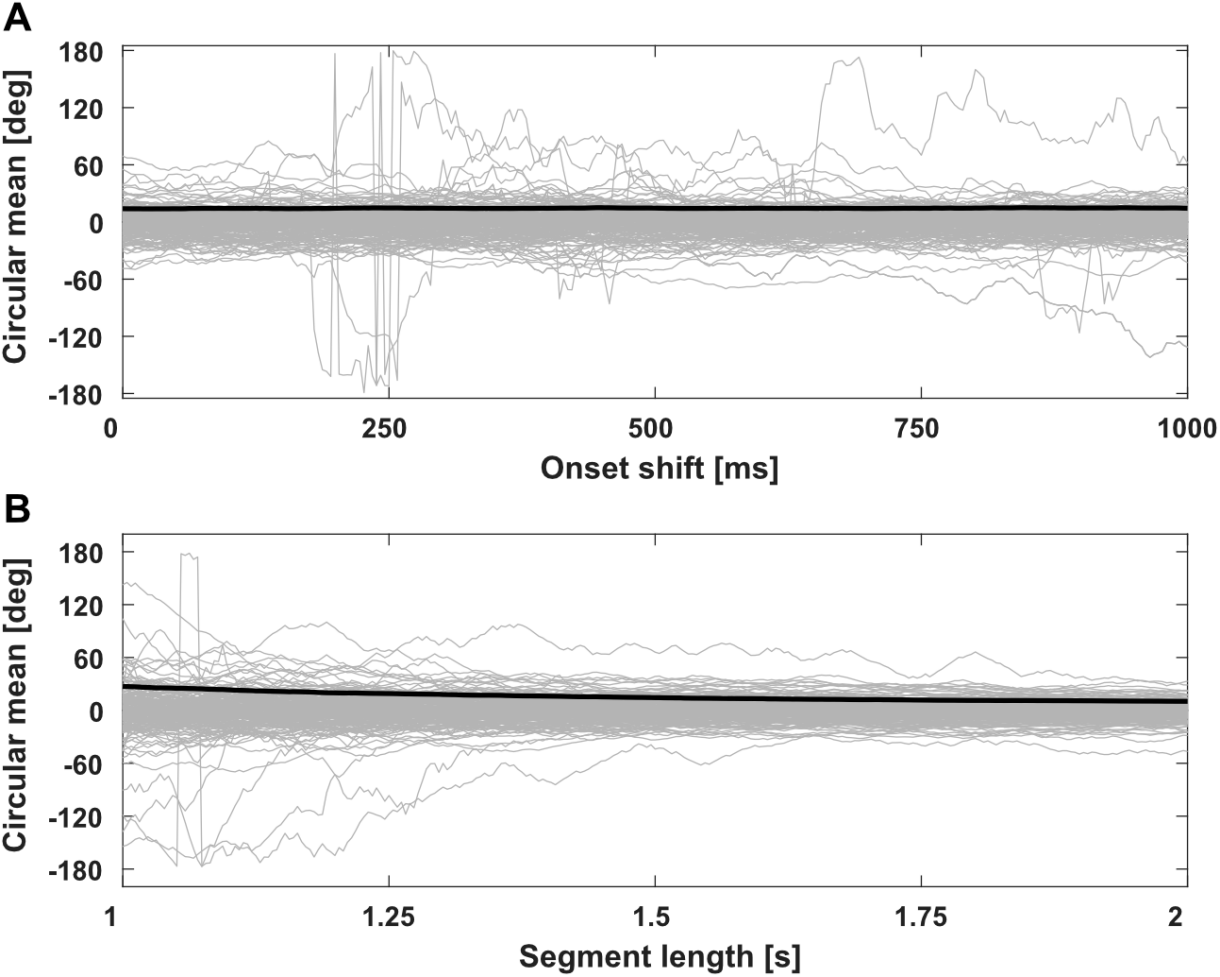
Variability of circular mean for continuous EEG signals (simulated data). (A) Estimated circular mean [deg] for individual simulated EEG signals over shifted epoch onsets; (B) estimated circular mean [deg] for individual simulated EEG signals over varying segment lengths. For methodological details seeFigure 1. Grey lines indicate individual simulated trials; thick black lines indicate standard deviations over 5000 simulated trials.

Circular mean values of continuous, simulated EEG signals varied considerably with small changes in the epoch onset (Fig. 3A) and epoch duration (Fig. 3B), especially for short epoch durations. The mean direction varied as much as 360 degrees (2π) for selected data segments. Variability of circular means reduced with increasing epoch lengths, whereas it remained largely constant with changing epoch onsets at fixed epoch lengths. This further suggests that mean directions of EEG signal segments are not well defined.

#### 3.1.2 Circular correlation estimates—trial average

**Fig. 4.**
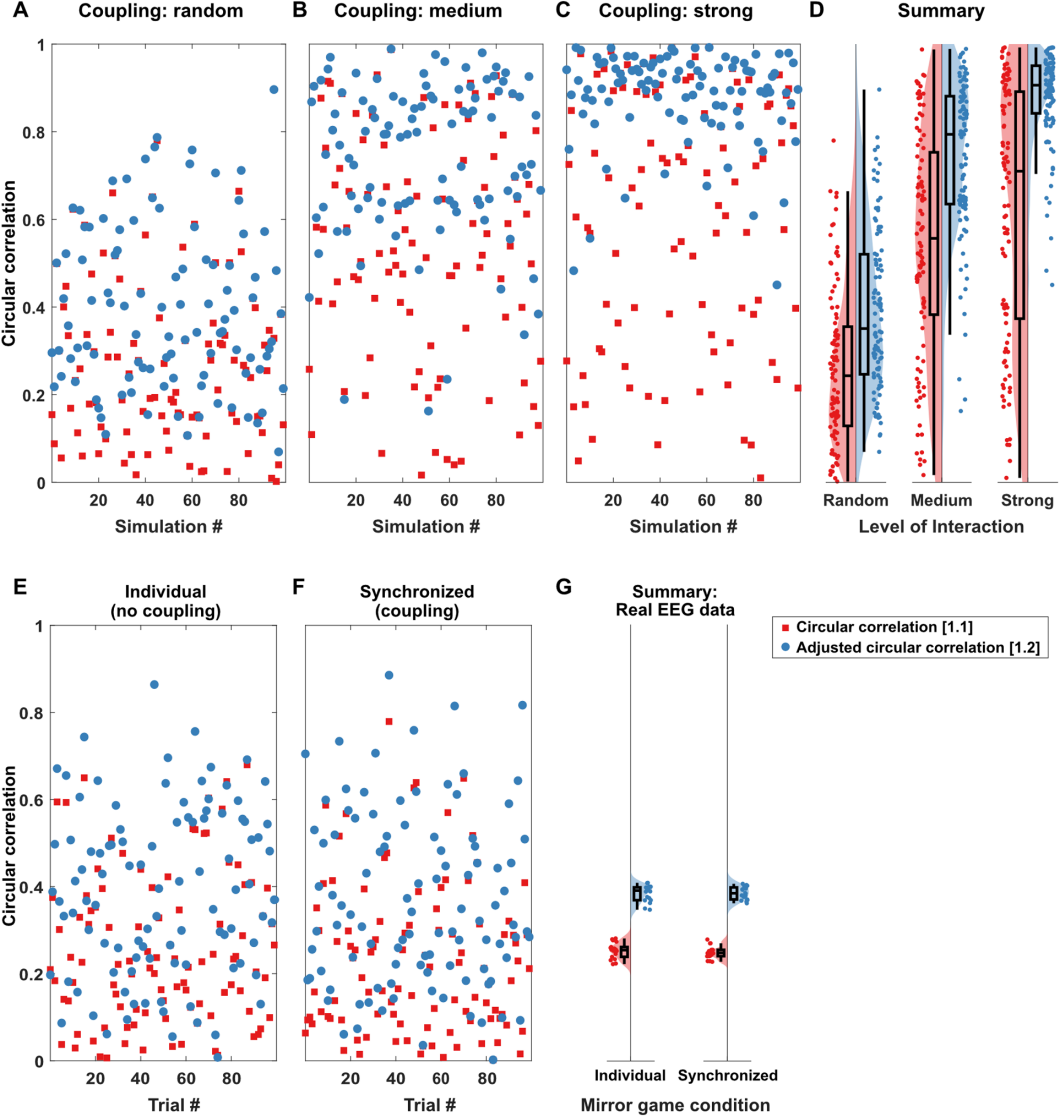
Circular correlation estimates by coupling level for simulated (top panels, A-D) and real EEG data (bottom panels, E-F). (A-D) Estimated circular correlation for 100 simulated trials each by equation, adjusted (blue) and not adjusted (red), and coupling level (random, medium, strong). Each dot represents a single simulated trial (A-C). (E-F) Single-segment circular correlation estimates for a single participant for individual and synchronized movement conditions in the mirror game (G) Group average estimated circular correlations on real data per dyad, for adjusted (blue) and unadjusted (red) circular correlation by coupling level.

First, we compared estimated circular correlations on individual trials for signals with no, medium, or strong coupling levels on artificial data (Fig. 4A-D). We obtained higher estimates for increased coupling levels (F(2,594) = 576.34, p < .001, η_p_^2^= 0.66), but also higher estimates for adjusted circular correlation (equation 1.2; adjusted for uniform data) compared to unadjusted circular correlations (equation 1.1; not adjusted) (F(1,594) = 176.49, p < .001, η_p_^2^= 0.23). There was an interaction between approach and coupling level (F(2,594) = 21.43, p < .001, η_p_^2^= 0.07); however, the effect of approach was observed at each coupling level (all p < .001), and estimates increased for each coupling level (all p < .001). Estimated circular correlations were 49.4% higher for data generated without coupling, 36.6% higher for data generated with medium coupling, and 41.7% higher for data generated with strong coupling.

For real EEG data (Fig. 4E-G), we observed a significant effect of approach (F(1,17) = 3790.70, p < .001, η_p_^2^= 0.99), with higher values for adjusted circular correlations, but no effect of coupling level (F(1,17) = 0.12, p = .734, η_p_^2^= 0.02), and no interaction effect (F(1,17) = 0.57, p = .459, η_p_^2^= 0.03). An analysis in pseudo pairs provided consistent results (seeSupplementary Material S5). Adjusted circular correlation estimates for individual trials correlated highly (r > 0.99) with PLV estimates at all coupling levels (seeSupplementary Material S3for details).

#### 3.1.3 Circular correlation estimates—effect of onset shifting

As shown above (Fig. 3), estimated mean direction varied with small shifts in epoch onset. Consequently, we investigated how the two approaches to estimate circular correlations, adjusted and non-adjusted for uniform distributions with not well-defined means, vary in terms of estimated circular correlation with small onset shifts.

**Fig. 5.**
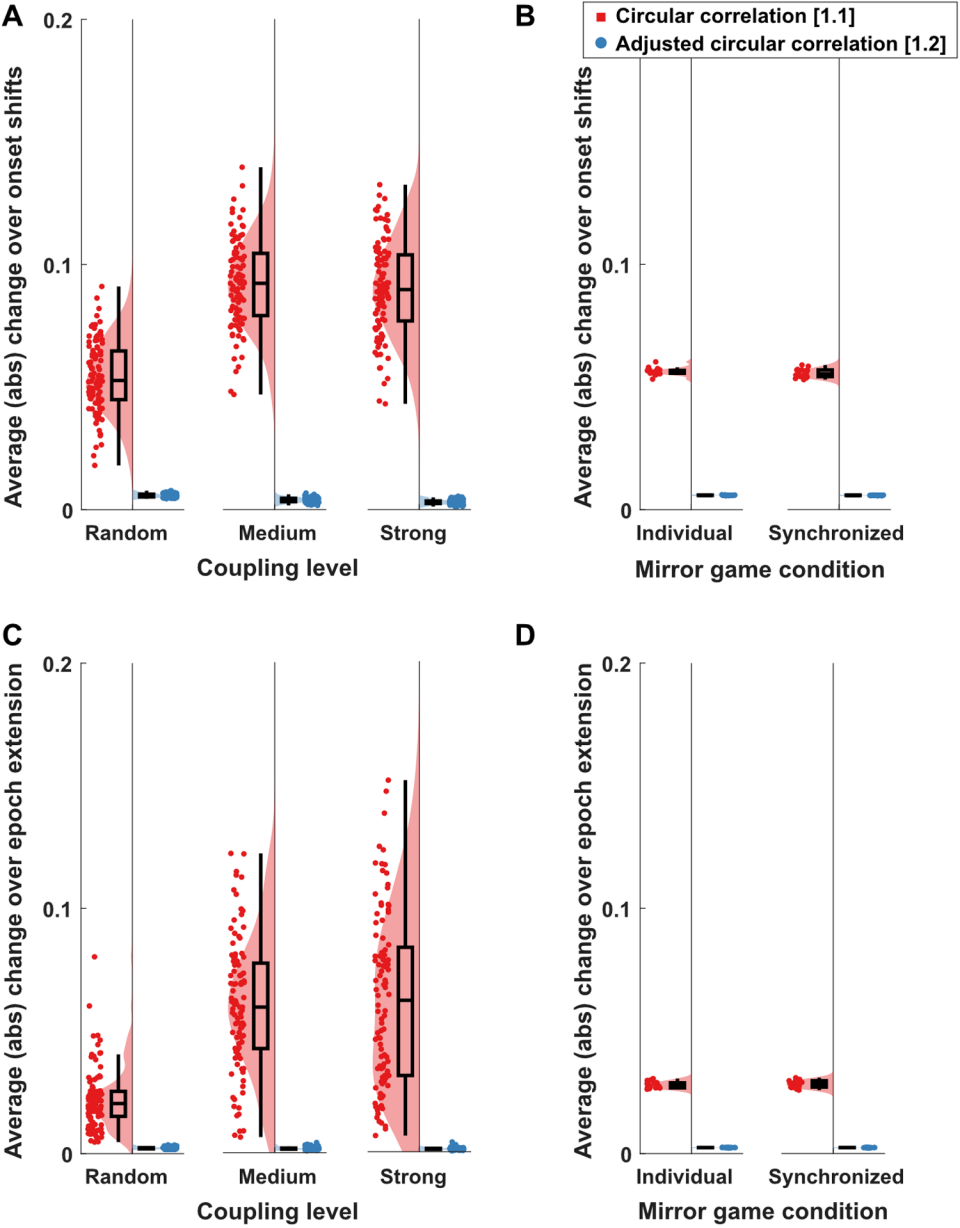
Effect of epoch onset shifts and incrementally extended epoch lengths on circular correlation estimates. (A-B) Effect of epoch onset shifts and (C-D) epoch length extension for simulated (left panels; A, C) and real (right panels; B, D) EEG data. Effects are measured as average sample-to-sample change of epoch onset or epoch length (by a single sample at a time at a sampling frequency of 256 Hz). SeeSupplementary Material S1for single-trial examples.

We observed a significant interaction between approach and coupling level (F(2,594) = 166.74, p < .001, η_p_^2^= 0.36), as well as significant main effects of approach (F(1,594) = 5493.39, p < .001, η_p_^2^= 0.90) and coupling level (F(2,594) = 130.53, p < .001, η_p_^2^= 0.31;Fig. 5A). Specifically, average change was higher forequation 1.1compared toequation 1.2for each coupling level (all p < .001), with a factor 9.41 for random, 23.38 for medium, and 29.53 for strong coupling levels. For unadjusted circular correlations, there was a significant increase in average change between random and medium coupling levels (t(198) = 16.39, p < .001; factor 1.69), as well as random and high coupling levels (t(198) = 14.70, p < .001; factor 1.66), but not between medium and strong coupling levels (t(198) = -0.77, p = 1; factor 0.98). In contrast, for adjusted circular correlations, average change decreased for higher coupling levels. Specifically, there was a lower average change for medium compared to random coupling levels (t(198) = 15.20, p < .001; factor 0.68) and a lower average change for strong compared to medium coupling levels (t(198) = 6.70, p < .001; factor 0.77), as well as between strong and random coupling levels (t(198) = 23.14, p < .001; factor 0.52).

For real EEG data (Fig. 5B), we observed significantly higher average changes for the unadjusted approach compared to the adjusted approach (F(1,17) = 30174.05, p < .001, η_p_^2^> 0.99), whereas there was no significant difference between the coupling levels (F(1,17) = 3.71, p = .071, η_p_^2^= 0.17) and no interaction between approach and coupling level (F(1,17) = 2.08, p = .168, η_p_^2^= 0.11).

#### 3.1.4 Circular correlation estimates—effect of epoch length

Similar to changes with shifts of epoch onset, estimated mean direction varied with small changes in epoch length. Consequently, we investigated how the two approaches to estimate circular correlations, adjusted and non-adjusted for uniform distributions with not well-defined means, vary in terms of estimated circular correlation with small changes to epoch length.

We observed a significant interaction between approach and coupling level (F(2,594) = 75.91, p < .001, η_p_^2^= 0.20), as well as significant main effects of approach (F(1,594) = 927.38, p < .001, η_p_^2^= 0.61) and coupling level (F(2,594) = 69.75, p < .001, η_p_^2^= 0.19;Fig. 5C). Specifically, average change was higher for non-adjusted circular correlations compared to adjusted circular correlations for each coupling level (all p < .001), with a factor 9.70 for random, 34.94 for medium, and 50.62 for strong coupling levels. For non-adjusted circular correlations, there was a significant increase in average change between random and medium coupling levels (t(198) = 13.06, p < .001; factor 2.72), as well as random and high coupling levels (t(198) = 10.70, p < .001; factor 2.80), but not between medium and strong coupling levels (t(198) = 0.39, p = .70; factor 1.03). In contrast, for adjusted circular correlations, average change decreased for higher coupling level. Specifically, there was a lower average change for medium compared to random coupling level (t(198) = 8.26, p < .001; factor 0.76), a lower average change for strong compared to random coupling level (t(198) = 16.77, p < .001; factor 0.54), and between strong and medium coupling level (t(198) = 6.84, p < .001; factor 0.71).

For the real EEG data (Fig. 5D), average changes over epoch length extensions were significantly higher for unadjusted circular correlations compared to the adjusted circular correlations (F(1,17) = 14329.39, p < .001, η_p_^2^> 0.99). We observed no significant difference between coupling levels (F(1,17) = 0.27, p = .608, η_p_^2^= 0.02) and no interaction between approach and coupling level (F(1,17) = 0.47, p = .503, η_p_^2^= 0.03).

### 3.2 Experiment 2: epoch length

#### 3.2.1 Effect of epoch length on IBS estimates

**Fig. 6.**
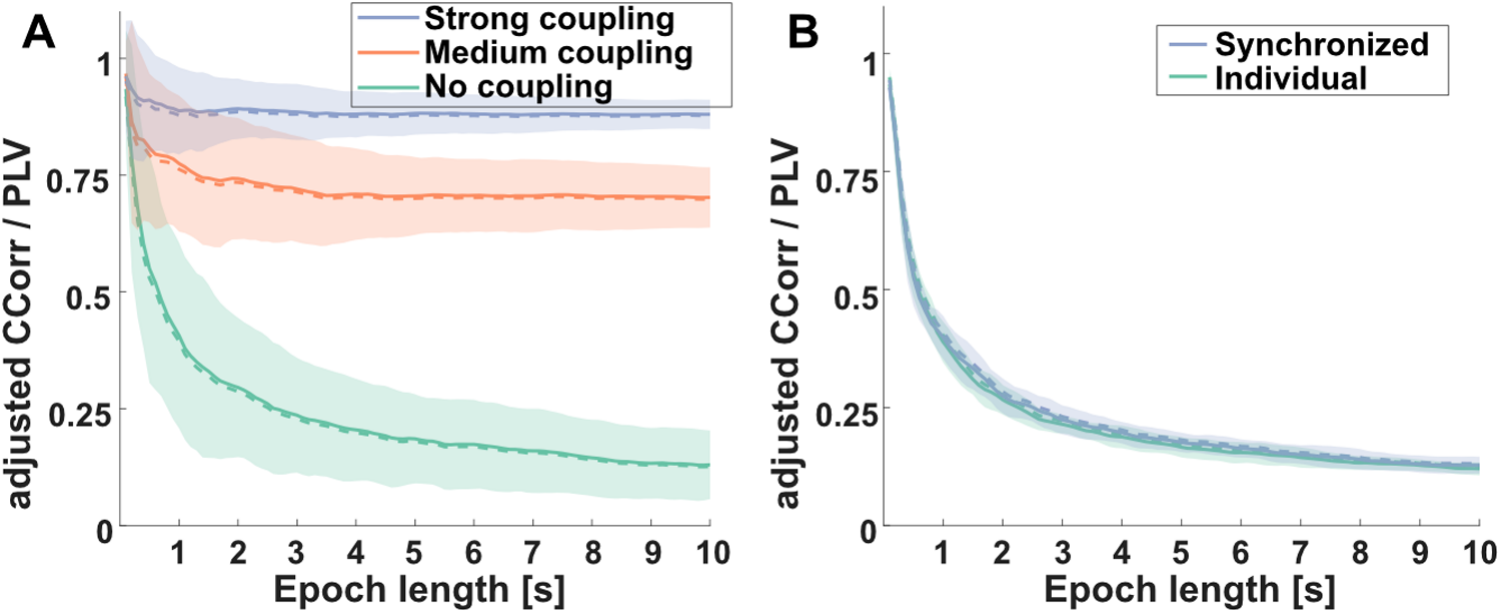
Effect of epoch length on inter-brain measures. (A) Results for adjusted circular correlation (solid lines) and PLV (dashed lines) for no, medium, and strong underlying coupling levels on simulated data. (B) Group average (N = 18) inter-brain synchronization estimates based on adjusted circular correlations (solid lines) and PLV (dashed lines) for individual and interactive trials (mirror game, 16 trials per actor pair). Shaded areas indicate standard deviations around the mean (for circular correlation estimates only). See alsoSupplementary Figure S4for a comparison of different frequency bands.

Visual inspection of the results (Fig. 6) suggests that estimates for inter-brain synchronization, both for PLV and adjusted circular correlations, decreased with epoch length, both for simulated (Fig. 6A) and real EEG data (Fig. 6B). Fitting an exponential function (b1 * exp(-b2*X) + b3) to the averages over all simulations confirmed that the “decay” of the high coupling condition was faster (b1 = 0.15, b2 = 0.41, b3 = 0.88) than the decay of the medium coupling condition (b1 = 0.34, b2 = 0.36, b3 = 0.70) and the no-coupling condition (b1 = 0.66, b2 = 0.06, b3 = 0.12). Based on visual inspection, the estimates reach a plateau at around 0.5 s for the high coupling data, at around 1 s for the medium coupling data, and at around 5 s for the no-coupling data. It should be noted that estimates for shorter epochs are systematically higher.

Within real EEG data, similar patterns were observed (Fig. 6B). Averaged over all pairs, estimates stabilized at epoch lengths of approximately 5–10 s, both for interactive and non-interactive trials. Average parameter estimates for the exponential fit for the individual condition were b1 = 0.70 ± 0.06, b2 = 0.07 ± 0.02, b3 = 0.12 ± 0.02, and for the interactive condition b1 = 0.70 ± 0.08, b2 = 0.07 ± 0.02, and b3 = 0.12 ± 0.02. No significant differences in parameters were observed between the interactive and individual conditions (all p > .10).

We repeated the same simulations with a higher frequency band (16–24 Hz, approximating beta frequency band), with mixed results. Estimates stabilized faster for the higher frequency band, especially for uncoupled and medium coupled signals (seeSupplementary Material S4). We also conducted complementary simulations with bursts instead of sustained inter-brain coupling (seeSupplementary Materials S2). In the case of such intermittent bursts of IBS, the ideal epoch length appears to be equal to the average duration of these bursts (Supplementary Fig. 2).

### 3.3 Experiment 3: power/signal-to-noise ratio

#### 3.3.1 Phase estimation error and SNR

**Fig. 7.**
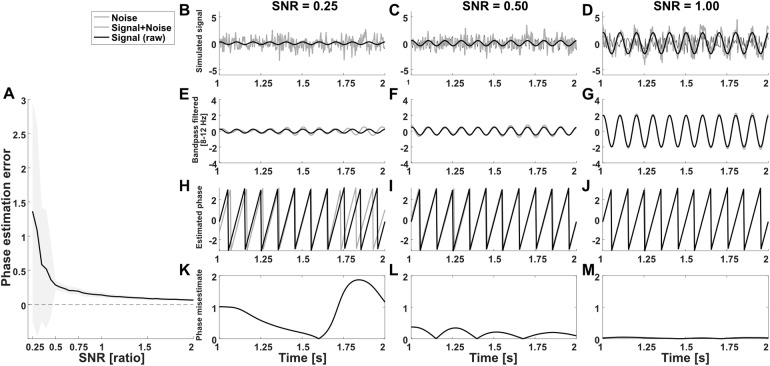
Effect of SNR on phase estimation error. (A) Phase estimation error is expressed as the RMS difference between estimated instantaneous phases (using Hilbert transform) of each trial while excluding possible edge effects, based on the combined (oscillation + noise) and the clean (oscillation only) generated signals. (B-M) Generated signal examples at different SNRs. (B-D) Simulated signal (raw+noise), including raw signal-and-noise component. (E-G) Band-pass filtered signal and signal components. (H-J) Estimated phase of raw (input) and mixed (raw+noise) signal. (K-M) mismatch between estimated phase of raw and mixed signal.

First, we investigated whether phase estimation error depends on signal-to-noise ratio. We observed a significant effect of SNR on phase estimation error (F(95,9504) = 50.32, p < .001, η_p_^2^= 0.33). Visual inspection of the results suggested decreasing estimation errors at higher SNR with stable estimates starting from SNRs of approximately 0.5 (Fig. 7A).

#### 3.3.2 IBS and relative noise levels

**Fig. 8.**
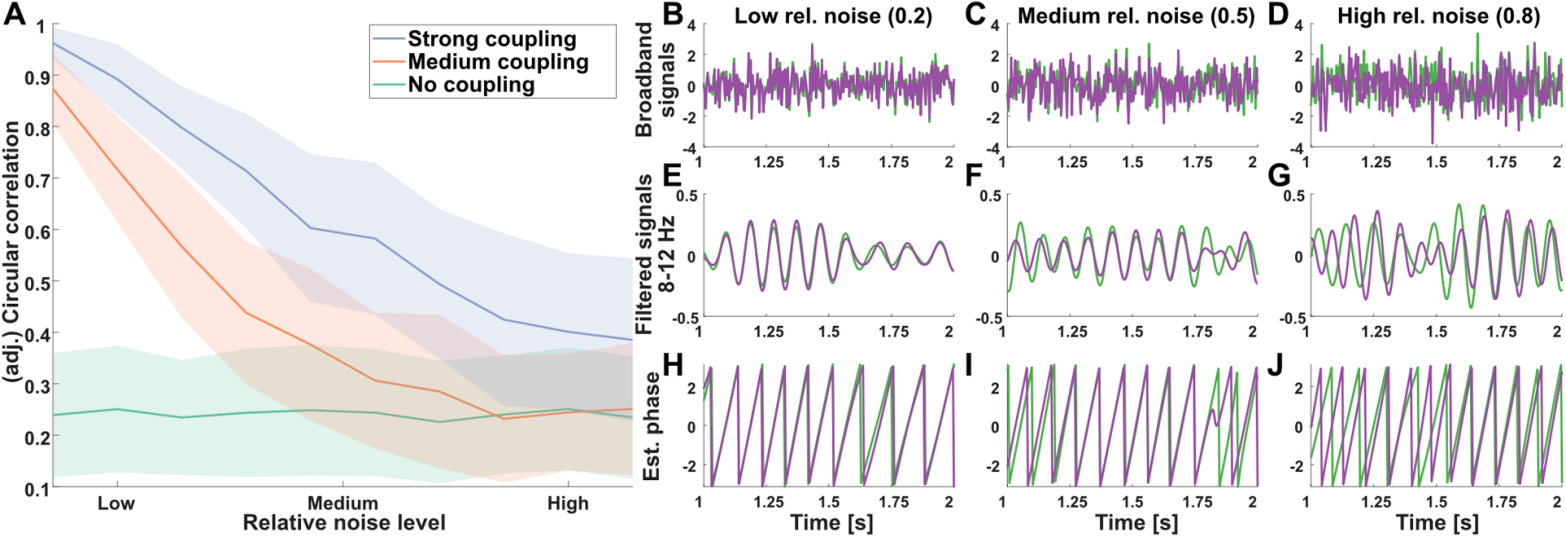
Effect of relative noise level on estimates of inter-brain synchronization (simulated data). (A) Effect of increasing level of relative noise on adjusted circular correlation for no, medium, and strong coupling data. (B-J) Detailed effect of relative noise level in data with strong stimulated coupling. For each noise level (in columns), (B-D) Raw signals for two actors; (E-G) band-pass filtered signals (8–12 Hz); (H-J) estimated phase per signal.

Simulation results with generated EEG data show that inter-brain synchronization estimates are reduced with increasing levels of relative noise, for coupling levels above zero. With no underlying coupling, IBS estimates remain approximately constant (Fig. 8). Specifically, we observed a significant interaction between coupling levels (no, medium, strong) and noise levels (low, medium, high) for adjusted circular correlations (F(4,891) = 248.85, p < .001, η_p_^2^= 0.53), with main effects for both coupling level (F(2,891) = 867.72, p < .001, η_p_^2^= 0.66) and noise level (F(2,891) = 935.20, p < .001, η_p_^2^= 0.68). Different circular correlation estimates were observed for different noise levels for strong coupling levels (F(2,297) = 1033.30, p < .001, η_p_^2^= 0.87) as well as medium coupling levels (F(2,297) = 1205.00, p < .001, η_p_^2^= 0.89), but not no coupling (F(2,297) = 0.16, p = .851, η_p_^2^< 0.01). Post-hoc pairwise two-sample t-tests revealed significant differences between strong and medium coupling levels for all relative noise levels, as well as for relative noise levels up to 0.7 [range 0.1–1.0] between medium and no coupling (all p < .05, Bonferroni corrected for 20 comparisons; 100 observations per cell, df = 198 for all comparisons). Visual inspection of the results (Fig. 8A) indicated decreasing estimates at higher levels of relative noise for signals with simulated coupling. In other words, as the SNR decreases, the synchronization between signals with stronger coupling decreases, and begins to resemble synchronization levels between uncoupled signals.

#### 3.3.3 Power and inter-brain measures in real EEG data

We compared adjusted circular correlation estimates in real EEG data (electrode C3) from a rest condition with estimates in a non-interactive condition of the mirror game, from the same dyads and recording session. Furthermore, we correlated average signal amplitude (based on the Hilbert envelope amplitude) with estimates of circular correlation. As assumed, alpha band power was higher in the rest condition compared to the movement condition (t(17) = 4.91, p < .001), confirming the occurrence of sensorimotor alpha-power suppression during the movement condition (factor 0.72; it should be noted that electrode C3 was chosen based on such an effect in a previous analysis of the same data, seeZimmermann et al. (2022)). No differences were observed in terms of adjusted circular correlation estimates between the (non-interactive) movement condition and the (non-interactive) rest condition (movement: mean ± sd 0.226 ± 0.012; rest: 0.232 ± 0.019; t(17) = 1.17, p = .259). Instantaneous power and adjusted circular correlation estimates were not correlated for the non-interactive movement task condition (z-transformed r = 0.003 ± 0.078 (MEAN±SD); t(17) = 0.14, p = .891); however, correlations were significantly higher than zero for the rest condition (z-transformed r = 0.082 ± 0.088; t(17) = 3.96, p = .001), and significantly higher than correlations in the movement condition (t(17) = 2.52, p = .022;Supplementary Fig. S6).

## 4 General Discussion

Our analyses of real and simulated EEG data have shown how phase-based inter-brain synchronization estimates may be greatly affected by arbitrary methodological decisions during different steps of data analyses. Specifically, we investigated the effect of arbitrary mean directions in computing circular correlation estimates of two signals with varying degrees of coupling, showing that circular correlation estimates are highly variable even during strong coupling due to large fluctuations in the circular mean direction. We propose a different implementation of the circular correlation coefficient using adjusted circular correlation, which adjusts for circular mean fluctuations.

Next, we showed how the use of short (1 s or less) epochs for the estimation of IBS, both using PLV and circular correlation, can result in highly inflated estimates, particularly in cases where there is no, or very weak, coupling between the signals. Longer (3–6 s or more) epochs are thus required to prevent such inflated estimates, as previously shown on functional connectivity estimates for single-brain EEG analyses (Fraschini et al., 2016;Miljevic et al., 2022). Finally, we have shown that IBS estimates become less reliable with lower signal amplitude, such as in conditions with stronger suppression of signal amplitude, for example, alpha or mu-suppression. Our data partially indicate a relationship between signal amplitude and IBS estimates, at least in the resting state condition. We unpack the results below, and provide recommendations for future research employing hyperscanning-EEG methods.

### 4.1 Circular correlation as measure for inter-brain synchronization

We systematically investigated the effect of approach regarding circular correlations for (dual) EEG data, based on real data as well as simulated EEG data with a known connectivity structure. We have shown that estimates of non-adjusted circular correlation in EEG data based onequation 1.1, that is, not adjusted for not well-defined/arbitrary mean directions, are highly variable and regularly underestimate circular correlation between coupled signals. Further, we have shown that the likely cause for these fluctuations, as well as underestimation, is the variability of mean direction with small changes in epoch length and epoch onsets, in the range of samples. Using adjusted circular correlations, followingequation 1.2, calculating circular correlations adjusted for arbitrary mean directions, in contrast, produces systematically higher values for circular correlation estimates, which do not fluctuate strongly with small changes in epoch length or onset. Values produced by adjusted circular correlations regularly form an upper bound for the estimates based on non-adjusted circular correlation (see examples inFigure 1and4;Supplementary Material S1).

Epoch length at the sample level, as well as on- and offsets, are arbitrary decisions that have to be regularly taken when conducting EEG analyses. Importantly, mutual adaptation or synchronization of neural activity between interacting partners should not be affected by the decision to analyze a time window shifted by a few milliseconds in an ongoing interactive process. Inter-brain estimates should be consistent along these parameters, both from a neurobiological point of view (as the behavior suggests an ongoing process) and from a statistical point of view (as there should not be substantial differences in data, for e.g., 254 or 256 samples). As suggested by Jammalamadaka (Jammalamadaka & SenGupta, 2001), mean directions for data with uniform distributions are not well defined. This is in line with our investigation showing large changes of mean direction with small changes in onset or duration of analyzed data segments (Figs. 1and3). Therefore, we recommend that for the purpose of EEG/MEG data, circular correlations as a measure of inter-brain synchronization should be estimated with adjustment for not well-defined mean directions of the data (adjusted circular correlation;equation 1.2), largely eliminating the influence of mean direction, and, therefore, of arbitrary decisions at the sample level with regard to epoch length and onset.

#### 4.1.2 Interpretational issues

We observed counterintuitive high levels of circular correlations for simulated EEG data with “no” coupling between the signals, with correlations around 0.3 (Fig. 4). One possible explanation for these high readings might be (at least in part) in the data preparation. Specifically, the data have been band-pass filtered according to narrow frequency bands (i.e., alpha, 8–12 Hz). In addition, circular correlations were estimated over data segments of 1 s. This raises the possibility that using short segments of data band-pass filtered to a narrow frequency range results in only a limited number of cycles, which is prone to produce more spurious coupling due to genuine similarities in EEG rhythms (given few cycles) between people (Burgess, 2013). While circular correlation coefficients have been proposed as a method that is actually less susceptible to this issue (Burgess, 2013), we show here that its original implementation may have other issues (i.e., with arbitrary mean directions), which when corrected for may be faced with the same overestimation of inter-brain synchronization as other connectivity methods, such as PLVs. A solution to this problem may be to use longer epoch lengths. This question is investigated insection 3.2and discussed insection 4.2.

One explanation for the disparity between our findings and those ofBurgess (2013)could be the different approach we took to generate simulated data. Given that EEG data are uniformly distributed, and hence do not have well-defined circular mean values, we simulated data with uniform distributions rather than using distributions with defined circular mean directions as inBurgess (2013). As we also see the same noisy estimates when calculating unadjusted circular correlation for real EEG data, we believe that the noisy estimates are due to the unadjusted approach rather than the method used to simulate data.

Another observation of analyses applied to the simulated data was that the difference between approaches decreases with increasing strength of coupling between the signals. Given the distribution of the data, this likely reflects a ceiling effect.

A third observation of the simulated data concerns the sample to sample estimate changes (Fig. 5), specifically, the interaction between approach (adjusted/unadjusted for arbitrary means) and the coupling level (high vs. low). For the adjusted approach, higher coupling levels resulted in lower sample-to-sample estimate changes, whereas for the unadjusted approach, higher coupling levels resulted in increased estimate changes. We think that, in case of the unadjusted approach, synchronization estimates take largely varying values between zero and the “true” coupling (see alsoFig. 4), depending on the estimated mean directions of the correlated signals. For the adjusted approach, in contrast, estimates of circular correlation for higher coupling levels are less affected by noise in terms of random/spurious “interactions” between signals, such as those we observed for the no-interaction simulations.

Finally, adjusted circular correlation estimates are remarkably correlated with PLV estimates, which raises the question of whether only one of these measures should be recommended for hyperscanning-EEG studies, for consistency purposes. Given that we have not systematically explored the differences between adjusted circular correlation and PLV, we encourage researchers to further explore this.

### 4.2 Effect of epoch length on IBS estimates (PLV and circular correlation)

Our analyses of real and generated EEG data showed that estimates for inter-brain synchronization, such as PLV and adjusted circular correlation, strongly depend on epoch length, in line with our expectations. In our data and simulations, where EEG data were band-pass filtered between 8 and 12 Hz (corresponding to the alpha frequency band), estimated inter-brain synchronization was higher for short epoch lengths of 1 s or less, and dropped sharply with extended epoch lengths, both for (generated) data with high and low/no implied coupling. Stabilization occurred after 1–5 s for generated data depending on the underlying coupling level, and approximately 5–10 s for real data (with unknown underlying coupling). These effects are comparable with data used to measure intra-brain connectivity, suggesting stabilization after 3–6 s (Fraschini et al., 2016). One reason for inflated IBS estimates for short epoch lengths may be because they contain only a small number of oscillation (Basti et al., 2022), which means they may be more prone to spurious coupling, making it more difficult to disentangle weak synchronization from randomly coupled signals.

Accordingly, optimal/minimal epoch length should depend also on the frequency band of interest. In fact, applying the same approach to generated EEG data band-pass filtered with 16–24 Hz (corresponding to the beta frequency band) resulted in stabilization at shorter epoch length (seeSupplementary Material S4), and lower estimates for generated signals without underlying coupling. These observations suggest that epoch length should be adjusted to the frequency band of interest, and systematic investigations are necessary to obtain optimal settings for all frequencies.

Based on these observations, we suggest that epoch lengths for estimation of inter-brain synchronization should ideally follow periods of behavioral coupling, but should be at a minimum 3 s long for data band-pass filtered to the alpha frequency band, and potentially longer following recommendations on intra-brain analyses fromFraschini et al. (2016)andMiljevic et al. (2022). Shorter epochs, as shown, can result in inflated and unreliable estimates, not only for signals that are coupled, but also for unrelated signals. This observation is particularly concerning as it can be assumed (Dumas et al., 2010;Lindenberger et al., 2009) that at least some inter-brain processes occur and manifest at shorter timescales than those recommended by previous research on intra-brain functional connectivity (Lachaux et al., 1999), and by us. One drawback, thus, of using longer epochs is that bursts of coupled activity or less stationary dynamical phenomena may be missed. This would be particularly true if IBS estimates were computed on entire non-epoched interactions, in case the entire 25 s segments, in which case the periods of phase coupling may be entirely smeared or missed. Our complementary simulations of bursting IBS instead of sustained coupling support this hypothesis. Overall, this calls for deeper investigation and characterization of the temporal evolution of IBS, and shows the limits of grand-averaging across tasks. A better sensitivity may require either more precise behavioral analyses of the social interaction or a more advanced way to detect those bursts of IBS.

### 4.3 Effect of power and signal-to-noise ratio on IBS estimates

Our analyses show that phase estimates become less reliable for experimental conditions where the EEG data have lower amplitude at the frequencies of interest (e.g., more mu or alpha suppression), despite a theoretical independence of amplitude and phase of a signal. This observation is in line with suggestions in the literature on intra-brain connectivity (van Diepen & Mazaheri, 2018), which suggests that phase estimates of ongoing oscillations are affected by the power modulations or concurrent evoked responses driven by task changes. Furthermore, our analyses show that IBS estimates computed using circular correlation coefficients and PLVs decrease with increasing levels of relative noise in the data.Figure 8shows that IBS estimates between strongly coupled signals with a high amount of noise, or low signal-to-noise ratios, resemble IBS estimates between non-interacting signals. An interpretation of this is that the less reliable phase estimates cause more noisy IBS estimates, which—on average—result in underestimation of the actual coupling between signals.

With respect to real EEG data, we show that resting-state trials with higher signal amplitudes yield higher IBS estimates than those with lower signal amplitudes, but this relationship is not present for movement data with no coupling. This is unexpected, given that there is no coupling between the participants, nor can they see each other or have any opportunity to exchange signals. One explanation for this is that higher signal amplitude results in higher estimates of inter brain synchronization; however, we only find a direct correlation between the signal amplitude and the IBS estimates in resting-state data. One reason for this may be due to larger variability in signal amplitude in the rest data compared to the movement data (seeSupplementary Material S6for details). This opens the possibility that reduced power (e.g., due to suppression of alpha oscillations over occipital or sensorimotor areas) at constant coupling levels may at least, in part, result in lower estimates, given higher (relative) noise levels.

Currently, there are no methods known to correct for the influence of noise, or spectral power differences at specified frequencies on phase estimates. Therefore, we suggest that a comparison of IBS between conditions or groups should always be accompanied by a close inspection of the corresponding frequency power. Furthermore, if a comparison suggests differences in IBS estimates between conditions with different levels of frequency power (e.g., due to increased alpha/mu suppression in one of the conditions), these IBS differences should be interpreted with utmost care, as the observed differences may be a consequence of reduced (or increased) relative noise at the same actual coupling level.

### 4.4 No observed IBS differences reported between individual and interactive conditions in single electrodes

We note here that for the real EEG data, we report no significant differences in IBS estimates between individual and interactive conditions. This should be interpreted with caution as we do not do a systematic statistical comparison between conditions, but merely compare synchronization between people’s single (and symmetric) electrodes (i.e., C3, chosen based on previous literature) in a single pre-defined frequency band, for the purpose of demonstrating how methodological decisions may influence IBS estimates. Given that IBS estimates in real data are generally low, and that differences in IBS estimates between conditions are generally of low effect size, it is thus not unusual that both interactive and non-interactive conditions alike yield similar and low IBS values. For better sensitivity, this is thus advised to use nonparametric cluster-based statistical testing (Ayrolles et al., 2021); this not only provides a straightforward way to address the multiple comparisons problem but also allows the integration of biophysically motivated priors in the test statistic (Maris & Oostenveld, 2007).

### 4.5 Conclusion

In this paper, we show how non-standardized methodological decisions that have to be made by researchers when analyzing two- or multi-person EEG data can greatly affect or distort phase-based estimates of inter-brain synchronization. We focus our investigation on methodological decisions regarding: arbitrary mean directions as well as epoch length and epoch onset/offset when estimating circular correlation coefficients; non-standardized epoch lengths; and the comparison of conditions with different levels of signal-to-noise ratios or signal amplitudes. It should be noted that the decisions investigated in this paper, and the potential issues that may occur during the analysis of hyperscanning-EEG datasets, are not exhaustive. There are likely other important methodological decisions that may also influence IBS estimates, which are not investigated in this paper. For example, the decision of which EEG reference to choose is not standardized, and previous research on intra-brain analyses shows that the choice of reference (e.g., common average reference, mastoids, REST, surface Laplacian) may have large effects on EEG results (Kayser & Tenke, 2010;Yao et al., 2005,^2019^), and, in particular, may distort phase, and hence connectivity estimates (Chella et al., 2016;Guevara et al., 2005;Shirhatti et al., 2016). We thus encourage further investigation of referencing, and other potential non-standardized methodological decisions, with respect to hyperscanning-EEG data. We hope that the results of this work contribute to the development of standardized hyperscanning-EEG methods, in an effort to increase validity and replication of inter-brain synchronization findings.

## Supplementary Material

Supplementary Material

## Data Availability

Data and code are available at the OSF repository:https://osf.io/zwnm5/.
