# Supplementary material for: Arbitrary methodological decisions skew inter-brain synchronization estimates in hyperscanning-EEG studies
Source: Imaging Neurosci (Camb). 2024 Nov 1;2:imag-2-00350. doi: 10.1162/imag_a_00350 (PMC12290587; doi:10.1162/imag_a_00350)
Supplement: Supplementary Material [file imag_a_00350-supp.pdf]

Supplementary materials

Supplementary material S1

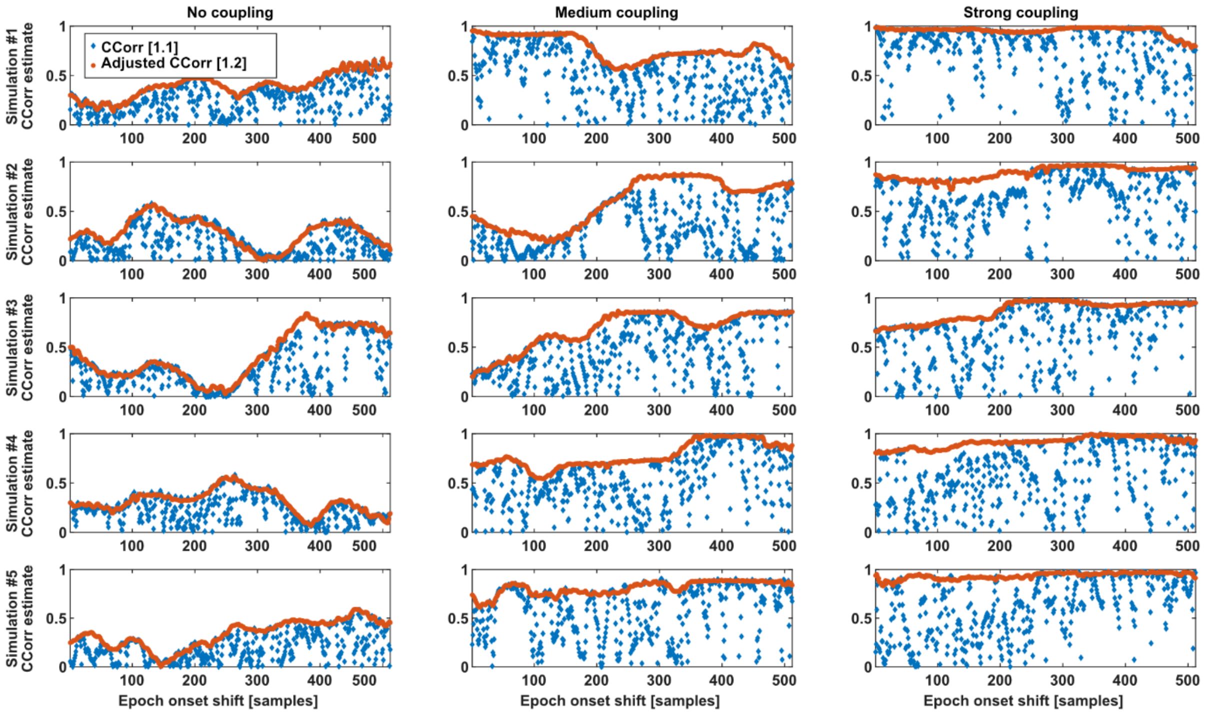

Supplementary Figure S1.1. Onset shifting effect examples on simulated data (see Figure 5). Effect of onset shift using equation 1.1 (not adjusted for uniform data) and equation 1.2 (adjusted for uniform data) to estimate circular correlations for random (left), medium (middle) and strongly (right) coupled signals. Displayed are the first 5 simulations for each condition.

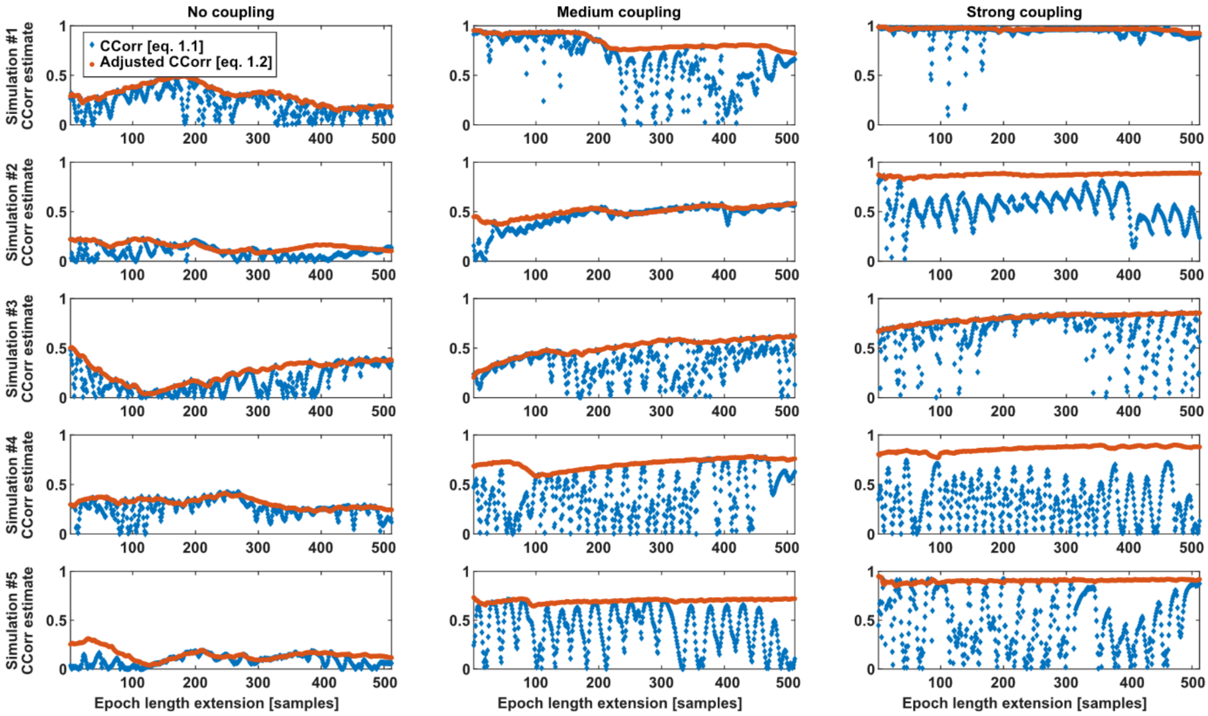

Supplementary Figure S1.2. Simulation examples for effect of epoch length **extension (see Figure 5).** Effect of epoch extension by single samples using equation 1.1 (not adjusted for uniform data) and equation 1.2 (adjusted for uniform data) to estimate circular correlations for random (left), medium (middle) and strongly (right) coupled signals. Displayed are the first 5 simulations for each condition.

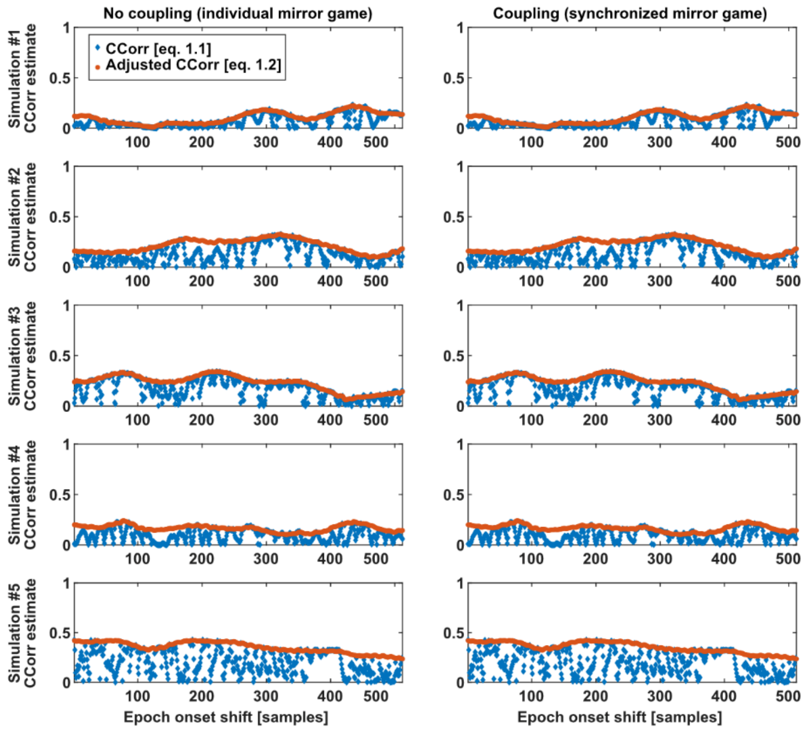

Supplementary Figure S1.3. Example trials for effect of epoch shifts on real EEG data (see Figure 5). Effect of onset shift using equation 1.1 (not adjusted for uniform data) and equation 1.2 (adjusted for uniform data) to estimate circular correlations for individual (left) and synchronized (right) mirror-game task condition. Displayed are the first 5 trials for each condition from a representative dyad.

Supplementary material S2

Inter-brain synchronization is not necessarily a sustained phenomenon. In order to further evaluate the impact of epoch duration, we also conducted complementary simulations with bursts of inter-brain synchronization. Using a linear mixing model like in the other simulations, we generated 500 pairs of 300-second oscillatory signals. However, instead of coupling them continuously, they were only coupled for a duration of 2 seconds every 20 seconds. We then ran the same inter-brain synchronization analyses, varying the epoch length from 1 second to 20 seconds, and compared two levels of inter-brain coupling (0.8 vs. 0.2). The Figure S2 shows how epoch durations change the effect size and highlights that 2 seconds epochs length — i.e. the burst duration — leads to the best sensitivity.

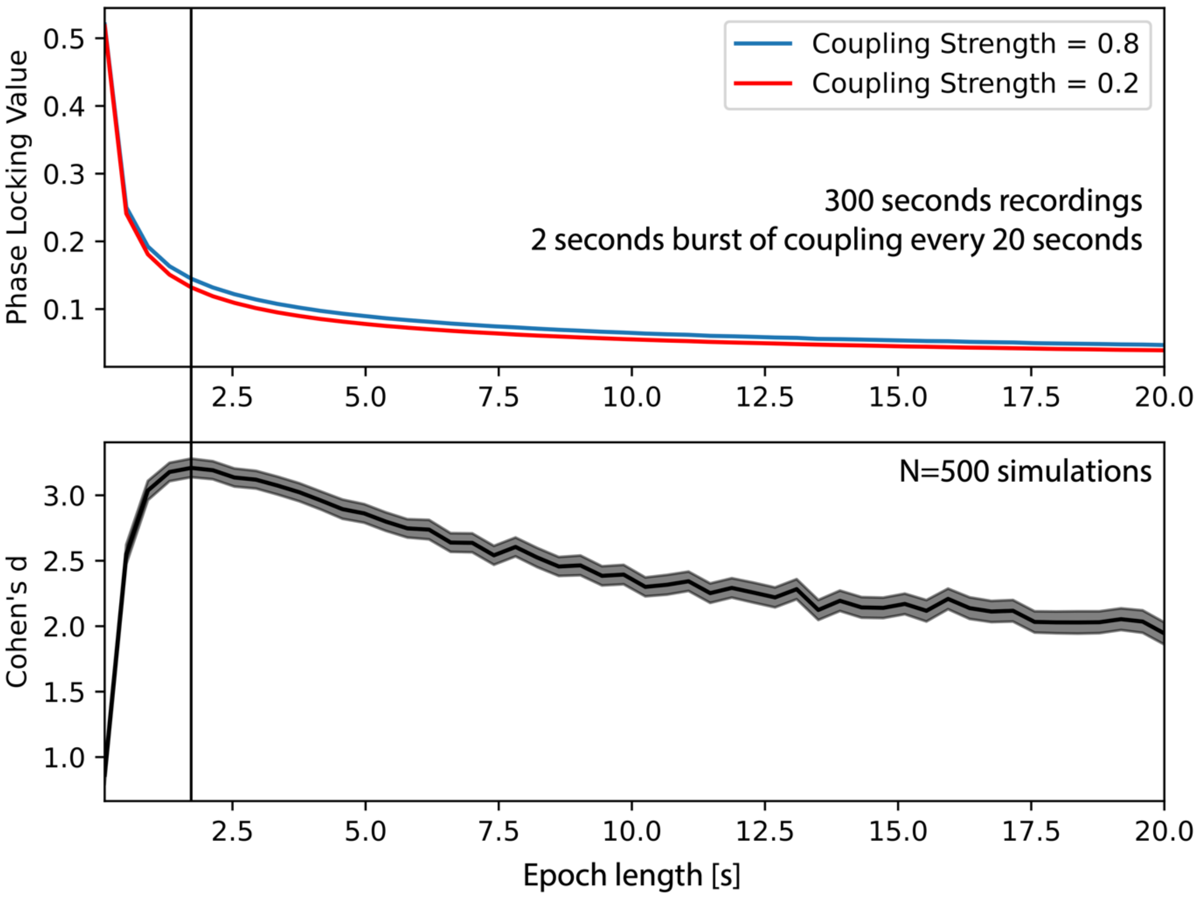

Supplementary Figure S2. Impact of epoch length on inter-brain synchronization burst detection. The top panel shows the average Phase Locking Values for a coupling strength of 0.2 (red) and 0.8 (blue). The bottom-panel shows the corresponding effect size of the difference in PLV between these two coupling strengths.

Supplementary material S3

Comparison of individual segment PLV and adjusted circular correlation estimates revealed high correlations at all coupling levels on simulated data (all r > .99; Figure S3 top). Similar high correlations (r > .9) have been observed for real data examples. Correlations between PLV and unadjusted circular correlation estimates were lower (coupling level random: r = .745; medium: r = .402; strong: r = .279, all p < .01; Figure S3 bottom).

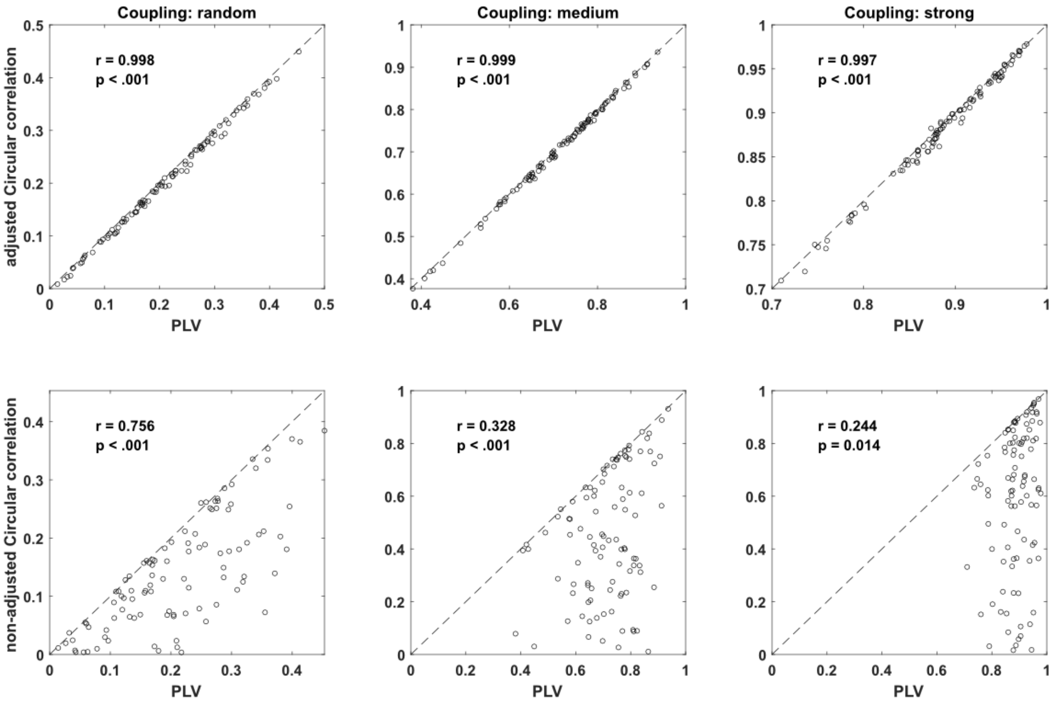

Supplementary Figure S3. Comparison of PLV and adjusted circular correlation (top) and standard circular correlation (bottom) for simulated data at random, medium and strong coupling level.

Supplementary material S4

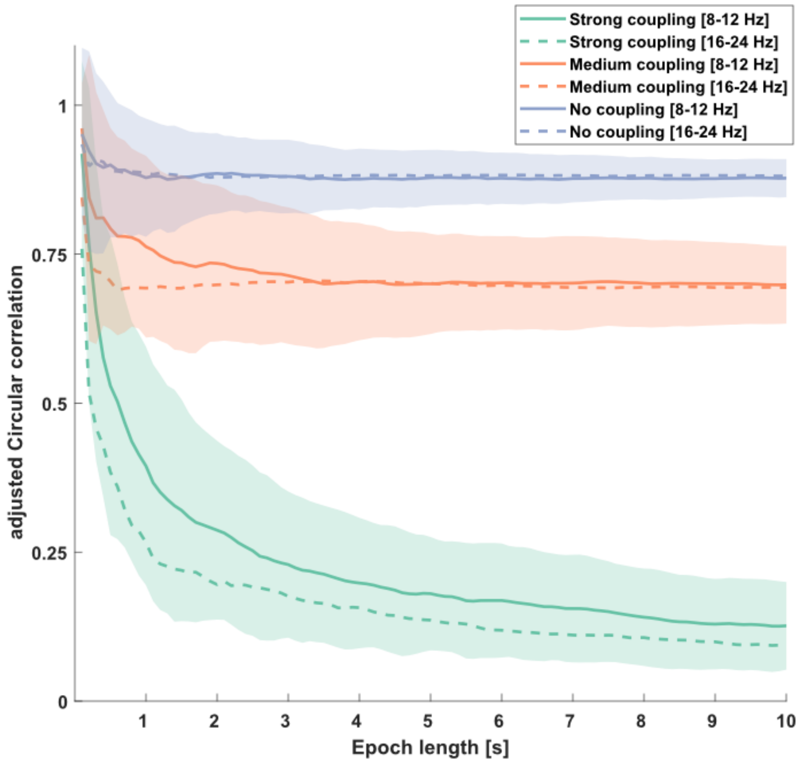

Supplementary Figure S4. Effect of epoch length on adjusted circular correlation estimates, for different band pass filtered frequency bands. Solid lines show alpha (8 – 12 Hz) band pass filtered inter-brain circular correlation (same as Figure 6). Dashed lines show approximate beta band data, band-pass filtered at 16 – 24 Hz. Visual inspection suggests faster stabilization of estimates with increasing epoch length (and thus, increasing number of oscillations, see discussion), especially for uncoupled and weakly coupled signals.

Supplementary material S5

Comparing circular correlation estimates using pseudo pairs provided similar outcomes as the analysis in valid dyads (section 3.1.2, Figure 4 E-G). In pseudo pairs, we observed a significant effect of approach ($F(1,17) = 1765.97$, $p < .001$), with higher values for the adjusted approach, but no effect of level of coupling ($F(1,17) = 0.03$, $p = .860$) and no interaction ($F(1,17) = 0.33$, $p = .573$).

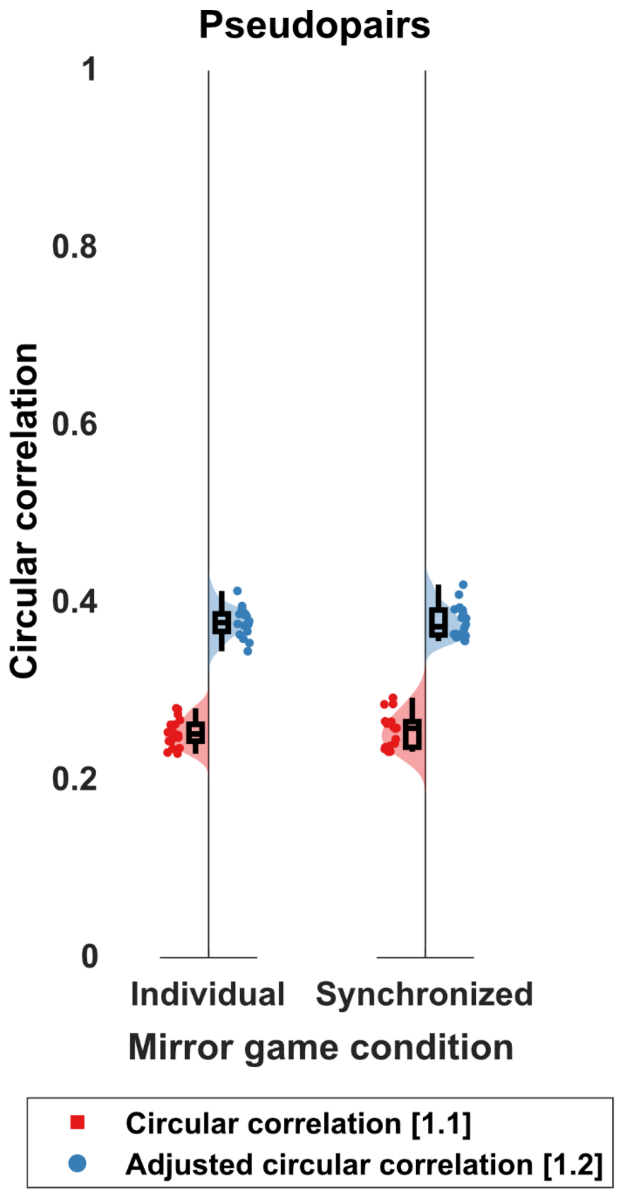

Supplementary Figure S5. Comparison of circular correlation estimates in pseudo pairs. Results are comparable to those in valid dyads (section 3.1.2, Figure 4).

Supplementary material S6

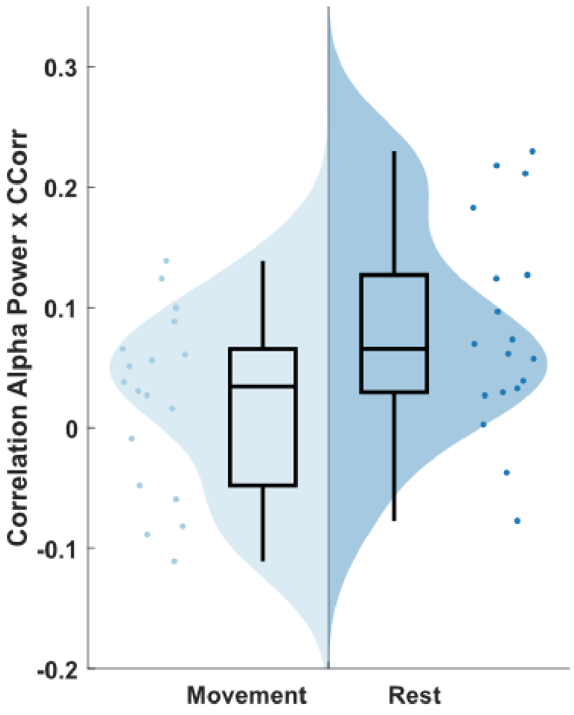

Supplementary Figure S6. Correlation between instantaneous power during the (non-interactive) movement task and rest condition and adjusted circular correlation estimates. Positive correlations were observed in the rest condition, but not the movement task. One reason for this may be due to larger variability in signal amplitude in the rest data compared to the movement data. Signal amplitudes were not only larger, but also had a larger range. Specifically, the standard deviations of individual signal amplitudes in rest data (0.633±0.254) were significantly larger than those in movement trials (0.207±0.046; $t(17) = 7.28$, $p < .001$).
